# Health-related quality of life of Korean older adults according to age, sex, and living arrangements: a cross-sectional study

**DOI:** 10.3389/fpubh.2023.1281457

**Published:** 2023-11-28

**Authors:** Horim A. Hwang, Hyunsuk Jeong, Hyeon Woo Yim

**Affiliations:** Department of Preventive Medicine, College of Medicine, The Catholic University of Korea, Seoul, Republic of Korea

**Keywords:** aged, oldest old, quality of life, residence characteristics, community survey

## Abstract

**Introduction:**

The number and proportion of older adults living alone is a significant issue. While the number of the oldest old people is also expected to increase, their health characteristics are poorly understood. This study aims to evaluate the health-related quality of life (HRQoL) of the oldest old people according to age, sex, and living arrangements.

**Methods:**

This study is based on the Korea Community Health Survey 2021. Among the survey's 229,242 observations, 73,617 observations aged 65 or higher were used for the analysis. The study participants were divided into 5-year age intervals (from 65–69 to 90+), sex, and living arrangements. The outcome variables are the EuroQol 5 Dimensions (EQ-5D) index score and the problem reporting rates of the five dimensions of EQ-5D.

**Results:**

The mean EQ-5D index scores were 0.896 at 65–69 and 0.741 at 90+. The mean EQ-5D index score decreased more rapidly as age increased. Women showed consistently lower mean EQ-5D index scores than men in all age intervals. The proportion of older adults living alone increased from 18.1% at 65–69 to 43.6% at 90+. The odds of reporting problems with anxiety/depression among older men living alone were estimated to be significantly higher than older men living with someone (aOR 1.22 95% CI 1.05–1.43). The odds of reporting problems in self-care and usual activity among older women living alone were estimated to be significantly lower than older women living with someone (aOR 0.88 95% CI 0.70–0.83 and aOR 0.88 95% CI 0.82–0.94)

**Conclusion:**

This study showed that older adults' HRQoL deteriorates as their age increases. Moreover, living alone may lead to different effects on older adults' HRQoL according to sex. More comprehensive studies and collaborative attention are needed to identify and provide customized care for older adults.

## Introduction

The proportion of people aged 65 or older in the global population is projected to increase from 10% in 2022 to 16% in 2050 (1). The increase in the proportion of older adults is prominent in Korea, where the proportion is expected to increase from 17.5% in 2022 to 40.1% in 2050 (2). As the number of older adults increases, the number of the oldest old is also expected to increase. The number of older adults living alone is consistently increasing as well. According to the Korean Statistical Information System (KOSIS), 18.8% of Koreans aged 65 or older were living alone in 2016, and the proportion of those living alone had increased to 20.8% in 2022 (3).

While there is no single agreed-upon process or mechanism of aging, it is generally accepted that age-related changes in metabolism and physiological function are important risk factors for several diseases and disorders (4, 5). It was found that factors such as age over 60 years old and women are associated with lower self-rated health (6, 7). Previous studies on living alone found its association with an increased risk of loneliness, depression, and mortality (8–10).

EuroQol 5 dimensions (EQ-5D) is a descriptive system often employed to assess the health-related quality of life (HRQoL) of the population of interest. In a sense, aging is a multifactorial ailment that has a cumulative influence on HRQoL. EQ-5D could be an effective measure of the health characteristics of older adults. However, there is still a knowledge gap in the health characteristics of a rising population group: the oldest old who live alone. Most of the studies using EQ-5D are focused on participants with specific diseases such as cancer (11). Few studies on the HRQoL of older adults were based on a mixture of institutionalized and community-dwelling older adults or were conducted on a small number of participants in specific regions (12–14). Institutionalization of older adults is known to influence their HRQoL negatively, and a small study population recruited from a specific region could be influenced by region-specific characteristics and may not correctly represent the nationwide health characteristics of community-dwelling older adults (15). Other studies on HRQoL of the nationwide population focused on relatively younger population groups and differences in HRQoL according to sex (7). To the best of the authors' knowledge, there was no study on the community-dwelling oldest old individuals that focused on their HRQoL and living arrangements. This study aims to fill that gap using nationwide survey data on the Korean population.

This study evaluates the HRQoL of older adults aged 65 years or older according to age, sex, and living arrangements. First, this study investigates the association between EQ-5D index scores and age. Second, this study explores the association between the EQ-5D index score and sex. Finally, this study evaluates the varying influence of living alone on the problem-reporting rate of the five dimensions of EQ-5D among older adults.

## Materials and methods

### Study design

This cross-sectional study used data from the Korea Community Health Survey 2021 (KCHS-21). Started in 2008, the KCHS is an annual nationwide survey organized by the Korea Disease Control and Prevention Agency, with support from 255 public health offices and 35 universities, that collects data from all regions of Korea to provide standardized regional-level public health statistics. KCHS-21 was conducted from 16 August 2021 to 31 October 2021. The target population is Koreans aged 19 or older as of 1 July 2021. The sample regions were extracted using probability proportional sampling, and the households were sampled using systemic sampling. On average, five households were interviewed per sample region. The interviewers for KCHS-21 were trained by public health offices and universities based on questionnaires and guidelines created by the Korean Disease Control and Prevention Agency. The interviewers visited households in person and conducted one-to-one computer-assisted personal interviewing (CAPI) with eligible household members. The survey data are collected between August and October of the survey year (16).

### Participants and data collection

**Figure 1:**
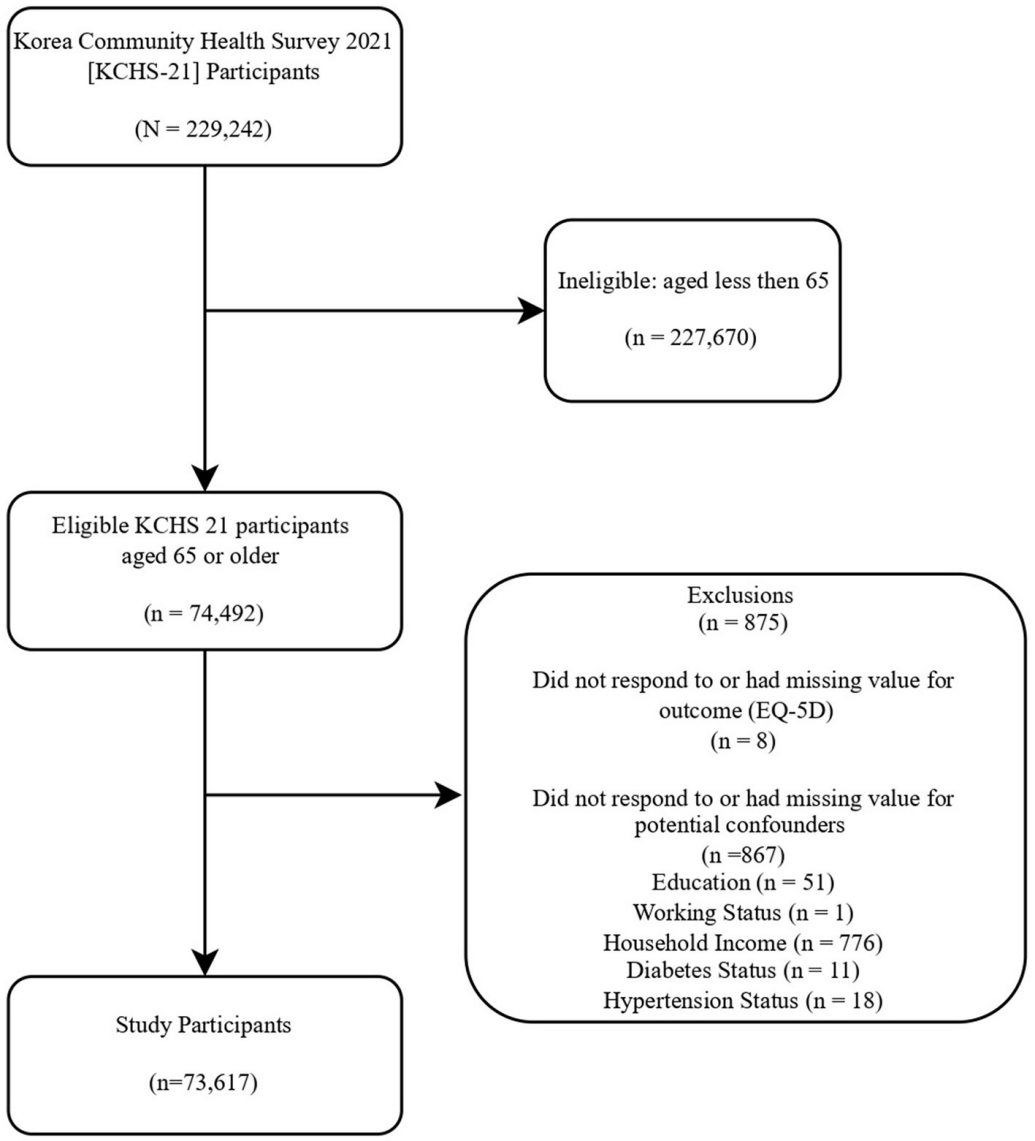
Flowchart of this study. Assessed KCHS-19 participants who did not respond to, or had no available data on, at least one of the EQ-5D dimensions, or potential confounders did not enter this study. Some ineligible participants did not respond to more than one category.

KCHS-21 participants constitute a population with a wide range of socioeconomic backgrounds and represent the entire population of Korea. Among KCHS-21′s 229,242 participants, 74,492 participants aged 65 or older were deemed eligible for this study. Eligible KCHS-19 participants who did not respond to, or had no available data on, at least one of the EQ-5D dimensions or potential confounders did not enter this study. Based on the above considerations, 875 participants did not enter this study due to non-response or missing values. Some of the ineligible participants did not respond to more than one variable. Eight eligible participants did not respond to or had missing values for EQ-5D dimensions. A total of 867 participants were excluded due to non-response or missing values for potential confounders. Finally, 73,617 participants entered this study. The response rate was 98.8% (Figure 1).

The participants were divided into a total of six age groups at 5-year intervals (65–69, 70–74, 75–79, 80–84, 85–89, and 90+). The age cutoff for the oldest old varied among previous studies. Some studies used 80 as the cutoff, while others used 85 or higher as the definition of the oldest old (13, 17). To encompass heterogeneous cutoffs from previous studies, we used 5-year intervals for age groups. For living arrangements, a study entrant was classified as living alone if the entrant reported no cohabitants in the household.

The outcome variables of this study are the EQ-5D index score and the five dimensions of the EQ-5D descriptive system. The EQ-5D is one of the most widely used self-rating instruments for measuring HRQoL. The EQ-5D descriptive system comprises five dimensions (mobility, self-care, usual activity, pain/discomfort, and anxiety/depression) with three levels: no problem, moderate problem, and extreme problem (18). EQ-5D dimensions were dichotomized based on problem reporting status. A moderate or extreme problem was categorized as yes for the problem reporting of the relevant EQ-5D dimension. Participants were stratified based on sex to analyze the EQ-5D index score and problem reporting rate. The authors used Korean-specific preference weight to calculate the EQ-5D index score (19). The five dimensions of EQ-5D are also categorized into physical dimensions (mobility, self-care, and usual activity) and mental dimensions (pain/discomfort and anxiety/depression). The N3 model was used to calculate the EQ-5D index score. The N3 model includes the “N3 term,” whose value is 0 or 1. The N3 term is 1 if a participant reported a level 3 or “extreme problem” in one or more EQ-5D dimensions and 0 if a participant reported no “extreme problem” in all EQ-5D dimensions. In addition to the preference weight value of each dimension of EQ-5D and N3 terms, the constant value was deducted if a participant reported any problems in one or more EQ-5D dimensions. The constant value was 0.050, and the N3 coefficient was 0.050 (19). An EQ-5D index score of 1 represents full health, 0 represents death, and a negative value represents a subjective health state worse than death.

To find out the independent effect of living alone on the problem reporting in mental dimensions, education, region, working status, household income, marriage status, and chronic disease were selected as potential confounding factors. Education was categorized as college or higher, high school, and middle school or lower. The region of living was divided into urban and rural. A study participant was categorized as employed for working status if employed full-time or part-time. Household income was categorized at the 2 million Korean Won (KRW) interval (approximately 1,500 US dollars in 2023): <2 million KRW per month (low), <4 million KRW per month (middle), and 4 million or higher KRW per month (high). Marital status was divided into three groups: married, bereaved, and other. KCHS-21 surveys the prevalence of two chronic diseases: diabetes and hypertension. Diabetes and hypertension status were measured based on the participants' self-reported diagnosis by doctors. If a study participant was diagnosed with at least one of the above two diseases, the entrant was classified as having a chronic disease category.

### Statistical analysis

A chi-square test was used to investigate the potential significant difference in general characteristics according to age intervals. Percentages were based on weighted numbers provided in KCHS-21 data. The Cochran–Armitage test for trend was used to estimate the increase in the problem reporting rate in the EQ-5D dimensions as age increases. Multiple linear regression was used to estimate the effect of age and sex on the mean EQ-5D index score. A two-way ANOVA was used to evaluate the interaction between EQ-5D dimensions and living arrangements on the problem reporting rate of the EQ-5D dimensions according to sex. Univariate logistic regression was used to verify the association between living arrangements and the problem reporting rates of the five EQ-5D dimensions when stratified by sex. Multivariate logistic regression was used to evaluate the independent effect of living alone on the problem reporting rates of the five EQ-5D dimensions according to sex after adjusting for potential confounders.

To explore the potential influence of COVID-19 on the association between living arrangements and five EQ-5D dimensions, we conducted a sensitivity analysis using multivariate logistic regression with KCHS-18 and KCHS-19 data. KCHS-18 and KCHS-19 are part of the same survey cycle as KCHS-21 and were conducted before the COVID-19 pandemic.

SAS 9.4 (SAS Institute Inc., Cary, NC, USA) was used for this study's analysis. The statistical significance level was set at a *p-*value of <0.05.

### Ethics statement

This study was approved by the Institutional Review Board of the Catholic University of Korea (approval No. MC23AZSI0020).

## Results

### General characteristics

Out of 73,617 study participants, 42,516 (55.5%) were women. The proportion of women increased across 5-year age intervals, from 54.1% at 65–69 to 68.2% at 90+. The number of participants decreased substantially at a 5-year interval, from 21,549 at 65–69 to 1,462 at 90+. The proportion of participants living alone increased from 21.8% at 65–69 to 43.6% at 90+. A similar pattern was observed for chronic diseases. A reversed pattern, where the proportion decreased from 65–69 to 90+, was observed for household income, working status, urban dwellers, and living with a spouse (Table 1).

**Table 1:** General characteristics of 73,617 older participants at a 5-year interval.

**Variables**	**Categories**	**Age**							
		**Total**	**65–69**	**70–74**	**75–79**	**80–84**	**85–89**	**90**+	
		***N** =* **73,617**	***n** =* **21,549**	***n** =* **18,281**	***n** =* **15,369**	***n** =* **11,726**	***n** =* **5,230**	***n** =* **1,462**	***p-*** **value** ^*^
		***n*** (%^*^)	***n*** (%^*^)	***n*** (%^*^)	***n*** (%^*^)	***n*** (%^*^)	***n*** (%^*^)	***n*** (%^*^)	
Sex	Men	31,101 (45.0%)	9,878 (46.9%)	8,165 (46.2%)	6,445 (45.4%)	4,515 (43.2%)	1,699 (35.4%)	399 (31.8%)	<0.001
	Women	42,516 (55.0%)	11,671 (54.1%)	10,116 (53.8%)	8,924 (54.6%)	7,211 (56.8%)	3,531 (64.6%)	1,063 (68.2%)	
Education level	College or higher	6,391 (13.3%)	2,807 (17.5%)	1,608 (12.8%)	1,029 (11.3%)	607 (9.3%)	285 (10.6%)	55 (8.8%)	<0.001
	High school	13,354 (23.7%)	5,836 (31.8%)	3,628 (25.0%)	2,137 (19.1%)	1,234 (15.5%)	429 (11.1%)	90 (9.6%)	
	Middle school or lower	53,872 (63.0%)	12,906 (50.7%)	13,045 (62.1%)	12,203 (69.6%)	9,885 (75.2%)	4,516 (78.3%)	1,317 (81.6%)	
Region	Urban	30,413 (72.9%)	9,939 (75.8%)	8,068 (74.7%)	6,156 (71.6%)	4,024 (67.0%)	1,733 (67.5%)	493 (67.5%)	<0.001
	Rural	43,204 (27.1%)	11,610 (24.2%)	10,213 (25.3%)	9,213 (28.3%)	7,702 (33.0%)	3,497 (32.5%)	969 (32.5%)	
Working status	Yes	30,211 (31.5%)	11,877 (46.8%)	8,368 (33.3%)	5,796 (25.2%)	3,277 (16.5%)	801 (8.1%)	92 (3.0%)	<0.001
	No	43,406 (68.5%)	9,672 (53.2%)	9,913 (66.4%)	9,573 (74.8%)	8,449 (83.5%)	4,429 (91.9%)	1,370 (97.0%)	
Household income (KRW/month)	High	6,835 (13.1%)	2,935 (17.6%)	1,568 (11.7%)	1,036 (10.0%)	773 (11.0%)	381 (11.8%)	142 (13.5%)	<0.001
	Middle	13,216 (21.8%)	5,861 (29.4%)	3,391 (21.9%)	1,998 (16.7%)	1,194 (13.9%)	584 (15.5%)	188 (19.4%)	
	Low	53,566 (65.1%)	12,753 (53.0%)	13,322 (66.4%)	12,335 (73.3%)	9,759 (75.4%)	4,265 (72.7%)	1,132 (67.1%)	
Marriage status	Married	45,946 (64.5%)	16,202 (74.5%)	12,740 (69.7%)	9,259 (62.8%)	5,705 (51.0%)	1,743 (36.4%)	297 (23.5%)	<0.001
	Bereaved	22,957 (27.7%)	2,962 (13.2%)	4,248 (22.1%)	5,516 (32.4%)	5,709 (45.4%)	3,374 (60.6%)	1,148 (75.3%)	
	Other	4,714 (7.8%)	2,385 (12.3%)	1,293 (8.2%)	594 (4.8%)	312 (3.6%)	113 (3.0%)	17 (1.2%)	
Chronic disease	Yes	45,705 (61.0%)	11,665 (53.1%)	11,414 (62.2%)	10,302 (67.7%)	7,871 (67.7%)	3,519 (67.9%)	934 (60.0%)	<0.001
	None	27,912 (39.0%)	9,884 (46.9%)	6,867 (37.8%)	5,067 (32.3%)	3,855 (32.3%)	1,711 (32.1%)	528 (40.0%)	
Living arrangements	Living alone	19,589 (21.8%)	3,901 (18.1%)	4,129 (22.6%)	4,441 (28.9%)	4,219 (36.0%)	2,262 (43.3%)	637 (43.6%)	<0.001
	Living with someone	54,028 (78.2%)	17,648 (81.9%)	14,152 (77.4%)	10,928 (71.1%)	7,507 (64.0%)	2,968 (56.7%)	825 (56.4%)	

Source: 2021 Korea Community Health Survey data of 73,617 eligible study participants aged 65 or older.

Percentages are based on the weighted number provided in the 2021 Korea Community Health Survey.

^*^p-value was calculated using the chi-square test.

### HRQoL according to age, sex, and living arrangements

**Figure 2:**
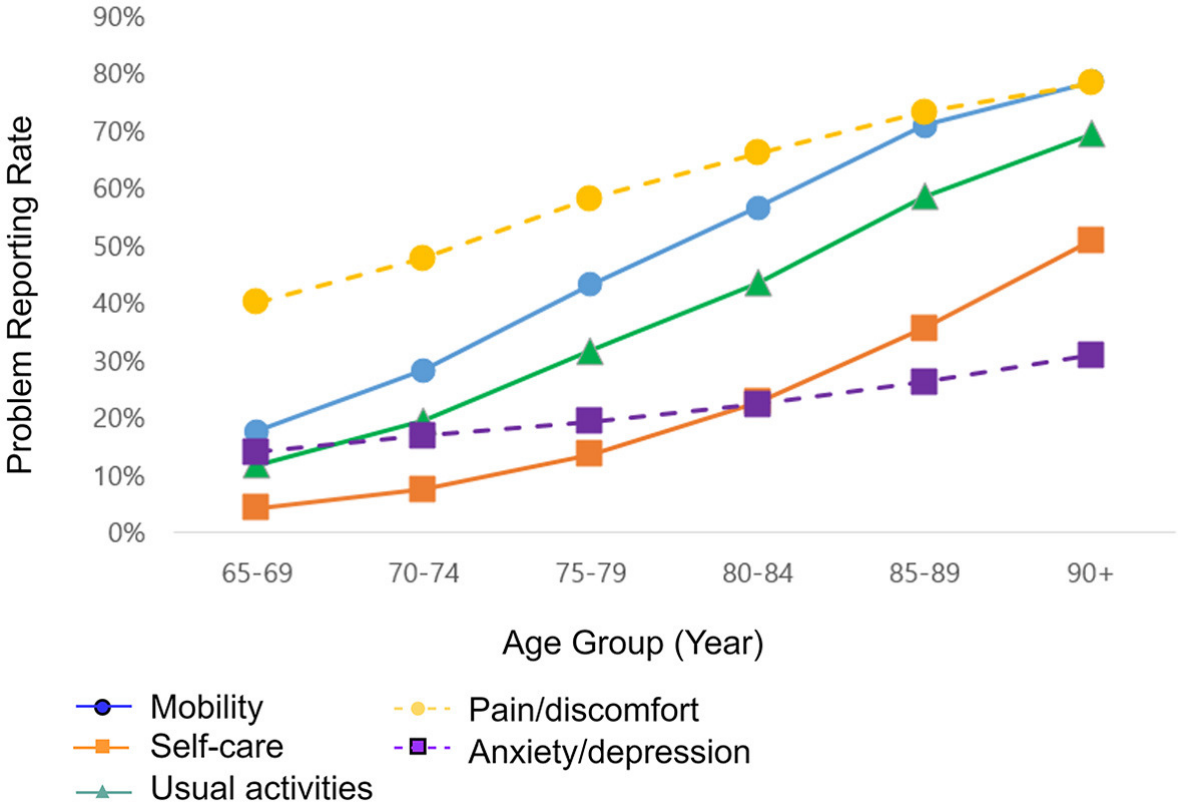
Problem reporting rate in five dimensions of EQ-5D of 73,617 older participants by age at a 5-year interval. Source: 2021 Korea Community Health Survey data of 73,617 participants aged 65 or older. A *P-*value of <0.001 for all five dimensions. The *P-*value was calculated using the test for trend.

The 65–69 age group showed the lowest problem reporting rate in all five dimensions of HRQoL (17.7% for mobility, 4.2% for self-care, 11.8% for usual activity, 40.2% for pain/discomfort, and 14.1% for anxiety/depression). There was a statistically significant trend toward an increase in the problem reporting rate at a 5-year interval for all five dimensions. For instance, in the anxiety/depression dimension, the problem reporting rate increased from 14.1% at 65–69 to 19.4% at 75–79 and to 30.9% at 90+ (Figure 2).

**Figure 3:**
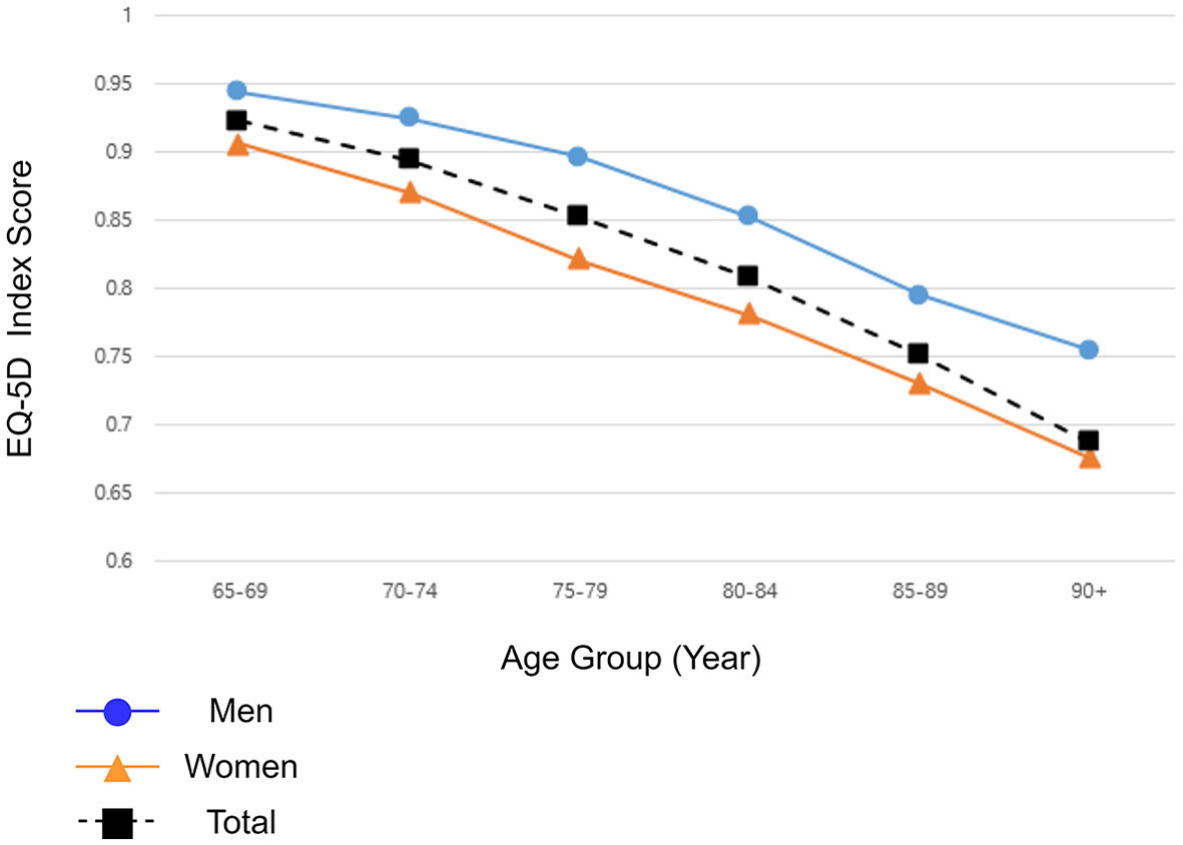
Mean EQ-5D index score of Korean older adults by age at a 5-year interval, stratified by sex. Source: 2021 Korea Community Health Survey data of 73,617 participants aged 65 or older. A *P-*value of <0.001 between the sex strata within the same age interval. The *P-*value was calculated using multiple linear regression.

The EQ-5D index score was highest at 65–69 (0.946). There was a general trend of decrease in the EQ-5D index score, and the older adults aged 90+ had the lowest EQ-5D index score (0.922 at 70–74. 0.885 at 75–79, 0.845 at 80–84, 0.792 at 85–89, and 0.741 at 90+). The EQ-5D index scores of older women were consistently low compared to men over all age groups (from 0.961 vs. 0.933 at 65–69 to 0.794 vs. 0.721 at 90+). The difference in the EQ-5D index score between the sex strata within the same 5-year interval was statistically significant in all age groups (Figure 3).

**Figure 4:**
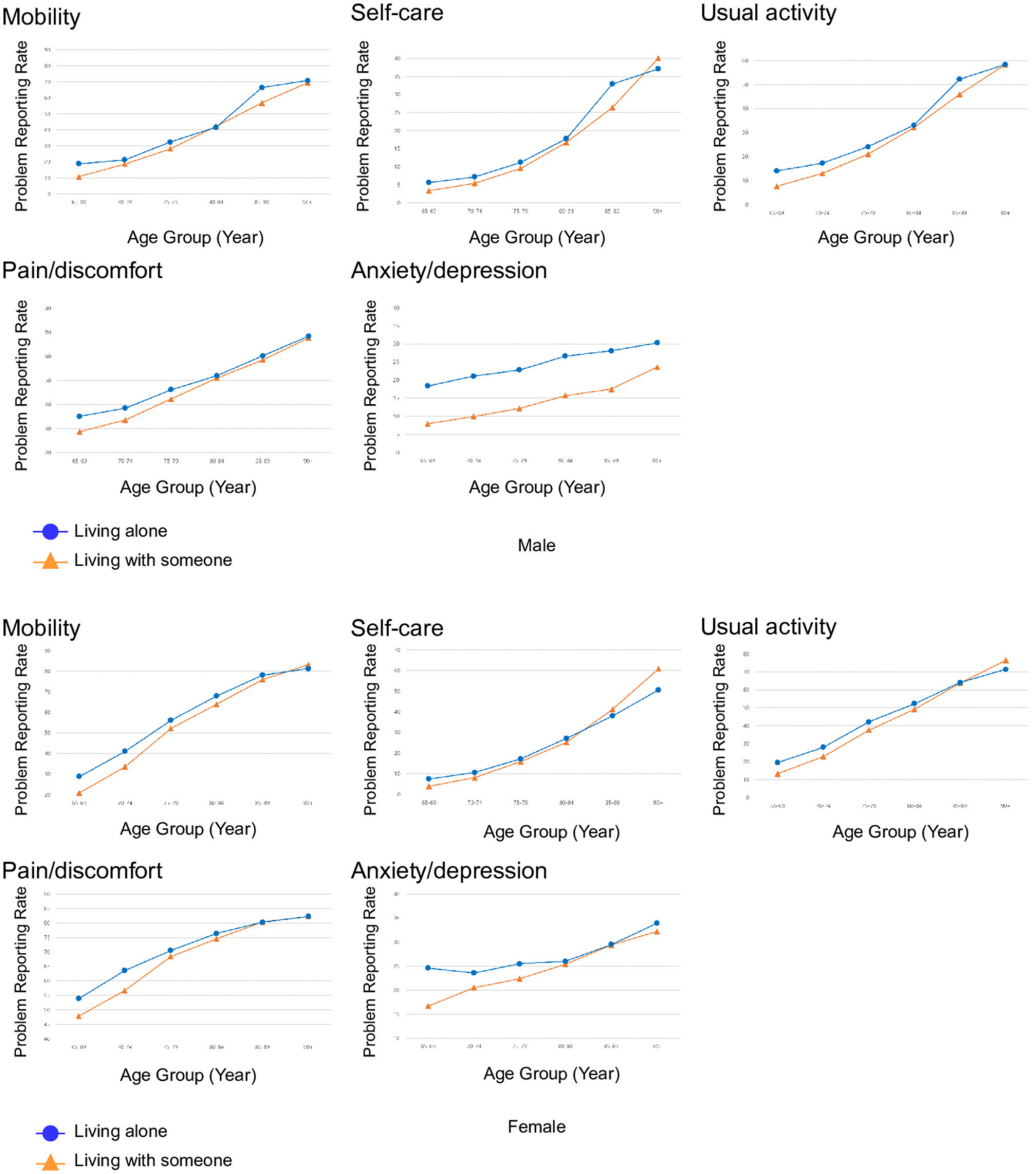
Problem reporting rate of moderate or extreme problems in five dimensions of EQ-5D of Korean older adults based on living arrangements, and age at a 5-year interval for mobility, self-care, usual activity, pain/discomfort, and anxiety/depression in men and women. Source: 2021 Korea Community Health Survey data of 73,617 participants aged 65 or older. A *P-*value of <0.001 for problem reporting rates in the anxiety/depression dimension between older men living alone and older men living with someone. The *P-*value was calculated using the chi-square test.

There was a significant difference in the problem reporting rate of anxiety/depression between the older men living alone and the older men living with someone. For all physical dimensions and a few mental dimensions, the differences in problem reporting rates between older adults living alone and older adults living with someone were not consistent for both sexes. The proportion of older adults reporting problems was higher or equal among those living with someone compared to those living alone at 85–89 in self-care, usual activity, pain/discomfort, and anxiety depression dimensions for older women, and at 90+ in mobility dimension for older women and mobility, self-care, usual activity, and pain/discomfort dimensions among older men (Figure 4).

There was no interaction between the anxiety/depression dimension and the living arrangements among the older men. An interaction effect was found between the three EQ-5D dimensions (self-care, usual activity, and pain/discomfort) and living arrangements for older men and between all EQ-5D dimensions and living arrangements for older women (Supplementary material 1).

The odds of reporting problems in the anxiety/depression dimension among the older men living alone were estimated to be significantly higher compared to the older men living with someone (aOR 1.22 95% CI 1.05–1.43). The older women living alone showed significantly lower problem reporting in the self-care and usual activity dimensions compared to the older women living with someone (aOR 0.77 95% CI 0.70–0.83 and aOR 0.88 95% CI 0.82–0.94). The older men living alone showed a similar likelihood of reporting problems in physical dimensions compared to the older men living with someone. The older women living alone showed a similar likelihood of reporting problems in the anxiety/depression dimension compared to the older women living with someone (Table 2).

**Table 2:** Odds ratios of problem reporting in EQ-5D dimension based on living arrangements, in (A) older men and (B) older women.

**A**	**Living with someone**	**Living alone**	
**EQ-5D dimension**		**Crude OR (95%CI)**	**Adjusted OR (95%CI)**
Mobility	Reference	1.31 (1.22–1.40)	0.96 (0.84–1.11)
Self-care	Reference	1.32 (1.19–1.46)	0.84 (0.70–1.02)
Usual activity	Reference	1.35 (1.25–1.46)	0.91 (0.78–1.06)
Pain/discomfort	Reference	1.21 (1.13–1.29)	1.04 (0.92–1.17)
Anxiety/depression	Reference	2.24 (2.06–2.43)	1.22 (1.05–1.43)
**B**	**Living with someone**	**Living alone**	
**EQ-5D dimension**		**Crude OR (95%CI)**	**Adjusted OR (95%CI)**
Mobility	Reference	1.84 (1.76–1.91)	0.96 (0.89–1.03)
Self-care	Reference	1.68 (1.59–1.77)	0.77 (0.70–0.83)
Usual activity	Reference	1.74 (1.67–1.82)	0.88 (0.82–0.94)
Pain/discomfort	Reference	1.51 (1.45–1.58)	0.96 (0.90–1.03)
Anxiety/depression	Reference	1.33 (1.27–1.39)	1.03 (0.96–1.11)

Source: 2021 Korea Community Health Survey data of 73,617 participants aged 65 or older.

Adjusted OR is adjusted by age group, education level, region, working status, household income, and chronic disease.

The sensitivity analysis with KCHS-18 and KCHS-19 showed a similar association between living arrangements and EQ-5D dimensions. The older men living alone were more likely to report problems in the anxiety/depression dimension than the older men living with someone (aOR 1.58 95% CI 1.45–1.70). The older women living alone were less likely to report problems in the self-care and usual activity dimensions than the older women living with someone (aOR 0.95 95% CI 0.90–0.99 and aOR 0.93 95% CI 0.89–0.96) (Supplementary material 2).

## Discussion

The current findings showed that the HRQoL of older adults varied according to age, sex, and living arrangements. For instance, the HRQoL of the oldest old people is similar to the HRQoL of patients with many well-known chronic diseases. Compared to the HRQoL of Korean patients with various diseases of varying severity levels, an average oldest old Korean individual in the 80–84 age range with an EQ-5D index score of 0.845 has a quality of life similar to that of a patient with partially controlled asthma (0.849). An average oldest old individual in the 85–90 age interval with an EQ-5D index score of 0.792 has a quality of life similar to a patient with severe sleep disturbance (0.807). An average oldest old over 90 years old with an EQ-5D index score of 0.741 has a quality of life similar to a patient with mild heart failure (0.793) or moderate COPD (0.742) (20).

The differences in mean EQ-5D index scores were 0.024, 0.037, 0.040, 0.053, and 0.051 over 5-year age intervals. The mean EQ-5D index score decreased more rapidly as age increased. The reported minimal clinically important difference (MCID) of EQ-5D-3L indices ranged from 0.028 to 0.08 (21–23). Based on the reported MCID range, there was a significant reduction in HRQoL as the age of the older adults increased. The EQ-5D index score decreased more rapidly as older adults grew older.

Compared to the older men of the same age interval, the older women showed consistently lower EQ-5D index scores and higher problem reporting rates of moderate or extreme problems in all five dimensions of EQ-5D. A similar result was observed in the HRQoL of the general population of England and the United States: women tend to report poorer HRQoL scores on nearly all dimensions than men (7, 24). A few biological mechanisms may explain the HRQoL difference between sexes. A higher prevalence rate of knee osteoarthritis among post-menopause women compared to men of similar age may explain a higher problem reporting rate in mobility, usual activity, and pain/discomfort. Women are known to have a greater risk of osteoarthritis development, and menopause has a multitude of effects on hormones, bone minerals, muscles, and tendons that, in turn, affect knee osteoarthritis (25, 26). Pregnancy may also contribute to the poorer HRQoL of women. Pregnancy complications such as preeclampsia and delivery methods such as cesarean section may lead to long-term consequences that could decrease HRQoL, but pregnancy's negative influence on HRQoL decades after delivery has not been assessed in previous studies (27, 28). The World Mental Health Survey of 15 nations showed that DSM-IV major depressive disorder occurs twice as often among women as men (29).

The proportion of older adults living alone increased from 18.1% at 65–69 to 43.6% at 90+. Among the oldest old men living alone who are 90+ years old, 70% reported problems in mobility, 35% in self-care, 60% in usual activity, 70% in pain/discomfort, and 30% in anxiety/depression. Among the oldest old women living alone who are 90+ years old, 80% reported problems in mobility, 50% in self-care, 70% in usual activity, 80% in pain/discomfort, and 35% in anxiety/depression. The relative rank of problem reporting rate of Korean older adults was similar to HRQoL patterns of the Hainan province Chinese community oldest old people, where the problem reporting rates in mobility and usual activity (approximately 45% for both mobility and usual activity in older men aged 91–95, and approximately 50% for both mobility and usual activity in older women aged 91–95), were highest and the problem reporting rate in anxiety/depression was lowest (approximately 20% for older men aged 91–95 and 10% for older women aged 91–95). However, older Koreans showed a higher problem reporting rate in nearly all EQ-5D dimensions and especially showed a twice or higher problem reporting rate in the anxiety/pain dimension (13).

The problem reporting rate in the three physical dimensions was higher or equal for both sexes at later age intervals of 80–84, 85–89, or 90+. There could be a reverse causation between problem reporting in the EQ-5D physical dimensions and living alone. Older adults prefer to maintain independence despite chronic conditions (30). An interaction effect was found between EQ-5D dimensions and living arrangements on the problem reporting rates of all five dimensions for older women. It is possible that older adults, especially older women with problems in physical dimensions, cannot maintain their independence as they age and resort to living with someone around the age of 80, 85, or higher.

The aOR point estimates of the effects of living alone on problem reporting in physical dimensions were lower than 1 for both sexes, suggesting the possibility that living alone is associated with lower odds of problem reporting in physical dimensions. Older adults with poor physical health are less likely than those with good physical health to maintain living alone status. A potential reverse causation between living alone and poor physical health may exist.

As for the independent effect of the living arrangements, the odds of reporting problems in the anxiety/depression dimension were 22% more likely among the older men living alone compared to the older men living with someone. The association between living alone and problem reporting in the anxiety/depression dimension remained significant among older Koreans before the COVID-19 pandemic era, confirming the robustness of this study's outcome. Anxiety/depression is a subjective value derived from a participant's perception of their current status, and living conditions influence the older adult's perception. Multiple factors were reported to be related to the increased risk of depression in older adults living alone. Social isolation and subjective physical and mental weakness have been reported to influence depression among older Korean men living alone (31, 32). Widowhood, low social quality of neighbors, and loneliness were associated with the increased risk of depression in older adults living alone in other Asian nations, regardless of sex (33, 34).

The living arrangements of an older adult, especially living alone, are an outcome of the preferences and resources an older adult has, along with the constraints they face as they age (35). Older adults may have different preferences according to their sex. According to the 2020 National Survey of Older Koreans, while older men prefer to live with their children more than older women do, most older adults who live with their children are actually women (36).

Among older men, older adults living alone showed a 1.5 times higher problem reporting rate in the anxiety/depression dimension than older adults living with someone across all age intervals. Living alone was independently associated with an increased likelihood of reporting problems in the anxiety/depression dimension for older men. Older men may show such associations because living alone is not their preferred living arrangement. On the other hand, older women showed no significant difference in problem reporting rate in the anxiety/depression dimension according to living arrangements, potentially because older women live alone by voluntary choice.

Older women living alone showed a lower problem reporting rate in physical dimensions than older women living with someone at the oldest old age intervals. Older women may resort to living with their children or other family members when their physical health deteriorates. On the contrary, older men living alone showed a higher problem reporting rate in physical dimensions than older men living with someone at the oldest old age intervals in physical dimensions. The oldest old men may have to maintain unwanted living arrangements—living alone—even if their physical health deteriorates, and maintaining unwanted living arrangements under poor physical health may lead to a greater gap in the problem reporting rate in the anxiety/depression dimension according to living arrangements.

Typical difficulties that older adults who do not live with their children suffer are lack of care during illness, anxiety, and loneliness; older adults with low household income showed higher reporting rates for the difficulties mentioned above (36). Older adults living alone tend to rely on institutional support like the social security system because they have a poorer financial support network than older adults living with someone (36). Older adults living alone are likely more vulnerable to the typical difficulties older adults face. Financial and medical support for older men living alone are needed. The anxiety/depression dimension of EQ-5D has been suggested as a screening tool for moderate to severe depression in a community setting (37). Depressive disorder is the most important risk factor for late-life suicide behavior, and men are associated with an increased risk of committing suicide (38). Financial and medical support for older men living alone with problems in the anxiety/depression dimension are needed.

In terms of old age, the reviews of recent literature pointed out that living alone, pain (chronic, psychological, and physical), and depression are associated with a higher risk of suicidal ideation and/or behavior (38–41). Older adults living alone with problems in one or more EQ-5D dimensions possess multiple risk factors associated with suicide, and they are likely underrepresented in KCHS-21. In summary, the oldest old adults aged 90 or older in this study could be particularly resilient older adults.

This study has a few limitations. This study is a cross-sectional study based on secondary data. The causative associations between living arrangements and EQ-5D dimensions could not be assessed. The authors could not evaluate when the EQ-5D dimensions changed or what events caused the change in the EQ-5D dimensions. In addition, we could not incorporate potential confounders, such as current medication or diseases other than diabetes and hypertension, which were not available in KCHS-21, into this study's analysis. KCHS-21 is based on the self-reports of the survey participants, and recall bias is a possibility. However, KCHS-21 is conducted by trained interviewers with a standardized interview method, and EQ-5D has been widely used in various studies. The validity and accuracy of the participants' responses are unlikely to be influenced.

Despite the above-mentioned shortcomings, this study holds a strength that previous studies did not offer. KCHS-21 is a nationwide survey that collects data from all regions of Korea, and its participants are a representative sample of the entire Korean population.

## Conclusion

This study has demonstrated that the increase in age, especially for the oldest old people, may lead to poorer HRQoL. It was found that older women tend to report poorer HRQoL compared to older men. This study also showed that living alone may have different effects on older adults according to sex. Older women living alone are less likely to report poor physical HRQoL than older women living with someone at the oldest old age intervals, while older men living alone were more likely to report poor mental HRQoL than older men living with some at all age intervals. More comprehensive studies and collaborative attention are needed to identify and provide customized care for older adults.

## Data availability statement

Publicly available datasets were analyzed in this study. This data can be found here: https://chs.kdca.go.kr/chs/main.do.

## Ethics statement

The studies involving humans were approved by Institutional Review Board of the Catholic University of Korea (Approval No. MC23AZSI0020). The studies were conducted in accordance with the local legislation and institutional requirements. Written informed consent for participation was not required from the participants or the participants' legal guardians/next of kin in accordance with the national legislation and institutional requirements.

## Author contributions

HH: Conceptualization, Data curation, Formal analysis, Visualization, Writing—original draft, Writing—review & editing. HJ: Supervision, Validation, Writing—review & editing, Conceptualization. HY: Conceptualization, Methodology, Supervision, Validation, Writing—original draft, Writing—review & editing, Visualization.
