# Supplementary material for: Health-related quality of life of Korean older adults according to age, sex, and living arrangements: a cross-sectional study
Source: Front Public Health. 2023 Nov 28;11:1281457. doi: 10.3389/fpubh.2023.1281457 (PMC10715451; doi:10.3389/fpubh.2023.1281457)
Supplement: Supplementary file 1 [file Table_1.DOCX]

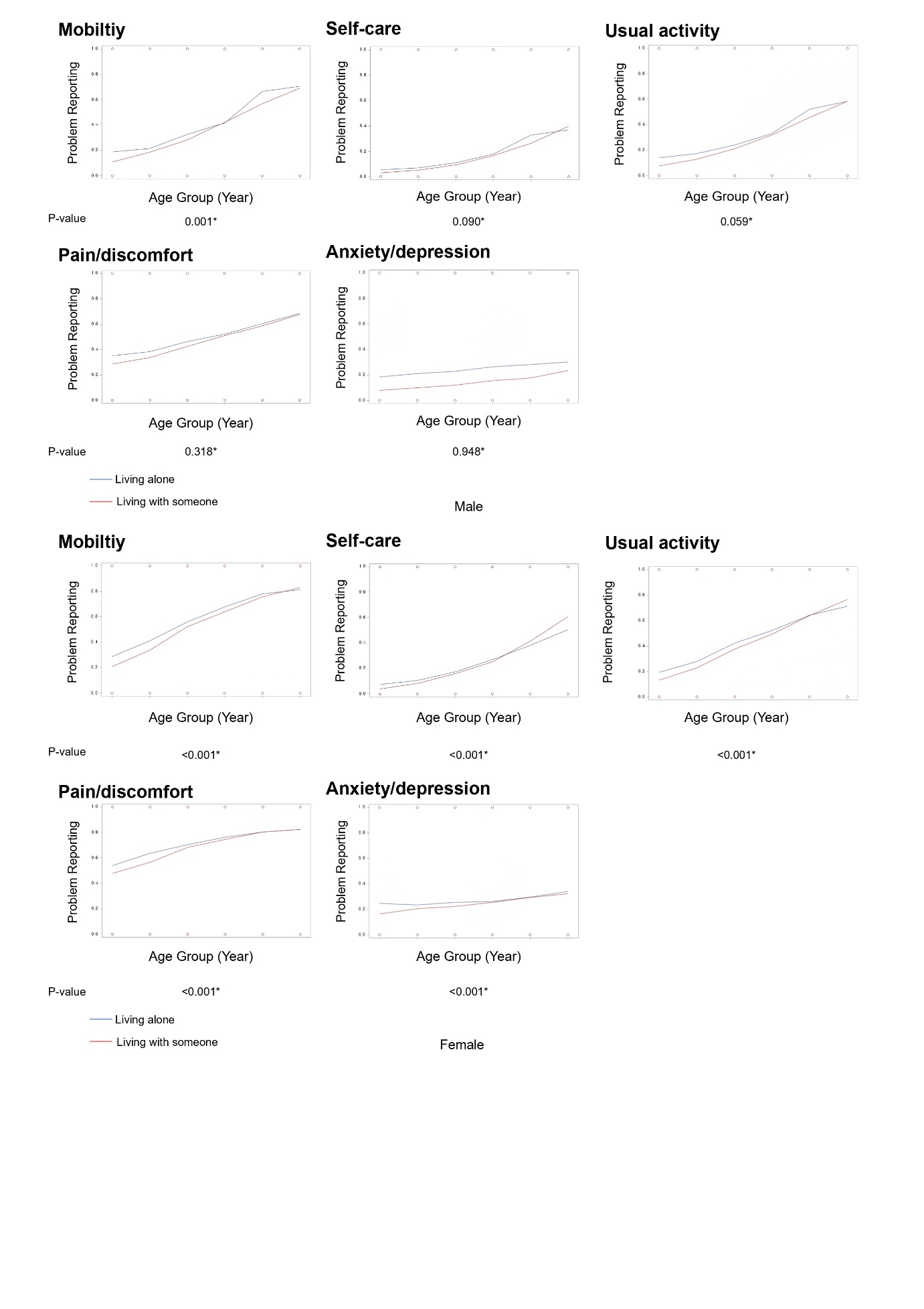
Supplementary Material 1. Analysis of interaction between age and living arrangements on the problem reporting rates of EQ-5D dimensions according to sex

Source: Author’s calculation using 2021 Korea Community Health Survey data of 73,617 eligible study participants

Notes: Two-way ANOVA was used for analysis. P-value for interaction between age and living status
