# Supplementary material for: Health-related quality of life of Korean older adults according to age, sex, and living arrangements: a cross-sectional study
Source: Front Public Health. 2023 Nov 28;11:1281457. doi: 10.3389/fpubh.2023.1281457 (PMC10715451; doi:10.3389/fpubh.2023.1281457)
Supplement: Supplementary file 2 [file Table_2.docx]

Supplementary Material 2. The odd ratios of problem reporting in EQ-5D dimension based on living arrangements in (A) older men and (B) older women using 2018 and 2019 Korea Community Health Survey data.

| **A** | Living with someone | Living alone | |
| --- | --- | --- | --- |
| EQ-5D dimension |  | Crude OR(95%CI) | Adjusted OR (95%CI) |
| Mobility | reference | 1.33(1.26-1.41) | 1.06(0.99-1.13) |
| Self-care | reference | 1.36(1.26-1.46) | 1.00(0.91-1.10) |
| Usual activity | reference | 1.32(1.25-1.40) | 1.01(0.94-1.09) |
| Pain/discomfort | reference | 1.25(1.18-1.31) | 0.98(0.87-1.10) |
| Anxiety/depression | reference | 1.95(1.83-2.08) | 1.58 (1.45-1.70) |
|  |  |  |  |
| **B** | Living with someone | Living alone | |
| EQ-5D dimension |  | Crude OR(95%CI) | Adjusted OR (95%CI) |
| Mobility | reference | 1.68(1.63-1.72) | 1.01(0.98-1.05) |
| Self-care | reference | 1.56(1.51-1.62) | 0.95(0.90-0.99) |
| Usual activity | reference | 1.53(1.49-1.58) | 0.93(0.89-0.96) |
| Pain/discomfort | reference | 1.40(1.36-1.44) | 0.93(0.87-1.00) |
| Anxiety/depression | reference | 1.22(1.18-1.26) | 1.01(0.96-1.05) |

Source: Author’s calculation using 2018 Korea Community Health Survey data of 69,559 eligible study participants and 2019 Korea Community Health Survey data of 72,828 eligible study participants.

Notes: Adjusted OR is adjusted by age group, education level, region, working status, household income, and chronic disease.
